# Establishment of a murine model of congenital toxoplasmosis and validation of a qPCR assay to assess the parasite load in maternal and fetal tissues

**DOI:** 10.3389/fmicb.2023.1124378

**Published:** 2023-02-27

**Authors:** Jéssica S. Souza, Priscila S. G. Farani, Beatriz I. S. Ferreira, Helene S. Barbosa, Rubem F. S. Menna-Barreto, Otacilio C. Moreira, Rafael M. Mariante

**Affiliations:** ^1^Laboratório de Biologia Estrutural, Instituto Oswaldo Cruz, Fiocruz, Rio de Janeiro, Brazil; ^2^Plataforma de PCR em Tempo Real RPT09A, Laboratório de Virologia Molecular, Instituto Oswaldo Cruz, Fiocruz, Rio de Janeiro, Brazil; ^3^Department of Biological Science, Border Biomedical Research Center, The University of Texas at El Paso, El Paso, TX, United States; ^4^Laboratório de Biologia Celular, Instituto Oswaldo Cruz, Fiocruz, Rio de Janeiro, Brazil

**Keywords:** *Toxoplasma gondii*, congenital toxoplasmosis, vertical transmission, outbred murine model, Swiss Webster, parasite load, quantitative real-time PCR

## Abstract

*Toxoplasma gondii* is the causative agent of toxoplasmosis, a disease that affects warm-blooded animals and one third of the human population worldwide. Pregnant women who have never been exposed to the parasite constitute an important risk group, as infection during pregnancy often leads to congenital toxoplasmosis, the most severe form of the disease. Current therapy for toxoplasmosis is the same as it was 50 years ago and has little or no effect when vertical transmission occurs. Therefore, it is urgent to develop new strategies to prevent mother-to-fetus transmission. The implementation of experimental animal models of congenital toxoplasmosis that reproduces the transmission rates and clinical signs in humans opens an avenue of possibilities to interfere in the progression of the disease. In addition, knowing the parasite load in maternal and fetal tissues after infection, which may be related to organ abnormalities and disease outcome, is another important step in designing a promising intervention strategy. Therefore, we implemented here a murine model of congenital toxoplasmosis with outbred Swiss Webster mice infected intravenously with tachyzoites of the ME49 strain of *T. gondii* that mimics the frequency of transmission of the parasite, as well as important clinical signs of human congenital toxoplasmosis, such as macrocephaly, in addition to providing a highly sensitive quantitative real-time PCR assay to assess parasite load in mouse tissues. As the disease is not restricted to humans, also affecting several domestic animals, including companion animals and livestock, they can also benefit from the model presented in this study.

## 1. Introduction

The apicomplexan parasite *Toxoplasma gondii* is one of the most successful pathogens of medical importance. It is estimated that one third of the human population is chronically infected with the parasite, and in some countries the seroprevalence can exceed 80% (Houdek, 2017). The success of their worldwide distribution is due in part to the multiple transmission mechanisms they have, which include the ingestion of undercooked meat containing cysts or contaminated water and vegetables containing oocysts (Dunay et al., 2018). If a woman becomes infected during pregnancy, the parasite can cross the placental barrier and gain access to the developing fetus, leading to congenital toxoplasmosis, the most severe form of the disease (Robert-Gangneux and Dardé, 2012).

Congenital toxoplasmosis is not restricted to humans. Companion animals, such as dogs and cats, and livestock, including ewes, pigs, goats, and cattle, are also infected through vertical transmission of *T. gondii* (Bresciani and da Costa, 2018). In livestock populations, the incidence of toxoplasmosis is high, and the transplacental transmission of the parasite is responsible for reproductive problems, such as spontaneous abortions, fetal malformations, premature, and stillborn animals, leading to high economic losses (Bresciani and da Costa, 2018). Therefore, not only humans, but also domestic animals can benefit from the work done with animal models of congenital toxoplasmosis.

The occurrence of congenital toxoplasmosis and the severity of the disease depend on when in pregnancy the woman is infected. The earlier a woman is infected, the less likely the parasite is to cross the placental barrier and infect the developing fetus, but the greater the severity of the infection should it occur. On the other hand, late infections increase the baby’s chance of becoming infected, but decrease the chance of complications, such as miscarriage and malformation (Dunn et al., 1999). Current treatment of pregnant women involves the administration of spiramycin, which is non-toxic and does not cross the placenta and is therefore used prophylactically to prevent vertical transmission of the parasite (Montoya and Remington, 2008), or a combination of pyrimethamine and sulfadiazine, when fetal infection is confirmed (Serranti et al., 2011). The effectiveness of such treatments, however, has not been strongly evidenced (Serranti et al., 2011). In this context, the development of new drug treatments to prevent transmission or treat the infected fetus is important.

Several models of congenital toxoplasmosis have been established over time, both with inbred and outbred mice (Pfaff et al., 2008; Pezerico et al., 2009; Senegas et al., 2009; Vargas-Villavicencio et al., 2016a,b, 2022; Sharif et al., 2018; Wang et al., 2018; Brito et al., 2020). In general, inbred mice are often preferred in comparison to outbred mice across biomedicine, since the assumption that they are less variable, more repeatable, and better defined than outbred stocks. However, recent evidences suggest that this is not necessarily true, and that outbred mice are actually more suitable for most biomedical research applications (Tuttle et al., 2018). Moreover, the susceptibility of inbred and outbred mice to infectious microorganisms may be quite different (Enriquez et al., 2020). Thus, the use of organisms with outbred genetic backgrounds, which highlights individual characteristics, in models of infectious diseases may better reproduce the heterogeneity of clinical outcomes observed in such diseases (Ribeiro-Romão et al., 2016).

Established mouse models of congenital toxoplasmosis present huge variability due to the use not only of different mouse strains, but also different parasite strains, parasite evolutive stages, parasite loads, inoculation routes, and time of pregnancy the infection of mothers occurs (Vargas-Villavicencio et al., 2016a), what make data obtained in those studies poorly comparable. The administration of tissue cysts through the oral route, for example, a strategy commonly used in mouse models of vertical transmission (Vargas-Villavicencio et al., 2016a), can lead to great heterogeneity in the outcome of the infection, since the number of bradyzoites within each cyst, regardless of their diameter, varies greatly (Watts et al., 2015). Therefore, inoculation of a known number of parasites is important to standardize the model. On the other hand, intraperitoneal administration of tachyzoites represents an artificial system that can present problems, since this route of inoculation drastically affects the outcome of the infection, either by affecting parasite replication or immune cell expansion, leading to worsen inflammation and increased mortality of mice (Meyer et al., 2013).

Vargas-Villavicencio et al. (2016b) used the intravenous route to inoculate a known number of tachyzoites of *T. gondii* in BALB/c mice to reproduce the peak of infection in humans, which occurs during the second trimester of pregnancy (Dunn et al., 1999). The intravenous administration of tachyzoites reproduces the hematogenous transplacental route, which is the mandatory mode of transmission of *T. gondii* in congenital infection (Carlier et al., 2012). The use of the low virulence type II ME49 strain was employed in that study (Vargas-Villavicencio et al., 2016b) and in a recent study by the same group where they evaluated infection during the first and last thirds of pregnancy (Vargas-Villavicencio et al., 2022). Congenital toxoplasmosis was mainly associated with type II strains in European, North American, and North African countries (Howe et al., 1997; Ajzenberg et al., 2002; Lahmar et al., 2020). Although this scenario does not apply to South America, especially Brazil, where there is high genetic diversity of *T. gondii* associated with congenital infections (Carneiro et al., 2013), the use of the ME49 strain as a model for the study of vertical transmission is important to establish a basis for comparative studies.

The quantification of parasites in different animal tissues in models of parasitic diseases is extremely important to determine the impact of the parasite load in experimentally infected animals. Real-time quantitative PCR is an essential tool for this purpose, as it presents great sensitivity, accuracy, and reproducibility in the quantification of tissue parasitism (Ribeiro-Romão et al., 2016). Therefore, in this study we (1) implemented a model of congenital toxoplasmosis combining the suitability of outbred Swiss Webster mice to study the heterogeneity of clinical aspects of the disease with the route of infection that best reproduces the maternal-fetal transmission of the parasite, and (2) standardized and validated a SYBR Green-based qPCR assay to assess the parasite load in maternal and fetal tissues in this model.

## 2. Materials and methods

### 2.1. Parasite maintenance

Parasites from the ME49 strain of *T. gondii* were obtained from the brains of C57BL/6 mice previously inoculated intraperitoneally with 50 cysts/animal, as described elsewhere (Guimarães et al., 2009). Briefly, tissue cysts are ruptured with an acid pepsin solution and free parasites are added to monolayers of Vero cells (ATCC^®^ CCL-81™). Two weeks later, cells are washed to remove free parasites and cell debris, fresh medium is added, and cultures are maintained for an additional 20 h. Fresh released tachyzoites in culture supernatant are harvested by centrifugation (1,500 × *g* for 10 min) and counted in a hemocytometer prior to use. To maintain their virulence, parasites are periodically passed *in vivo*.

### 2.2. Animals

Eight to ten weeks old outbred Swiss Webster (SW) mice (*Mus musculus*) obtained from the Institute of Science and Technology in Biomodels (ICTB/Fiocruz) were used in the study. The animals were kept in ventilated racks (Alesco, Brazil) equipped with a controlled ventilation system (10–20 air changes per hour) in an animal facility at Instituto Oswaldo Cruz (IOC/Fiocruz). The mice were housed in polypropylene cages (Alesco, Brazil) measuring 490 mm × 340 mm × 160 mm under a 12-h light:dark cycle at a temperature of 21 ± 2°C, relative humidity of 40–60%, with *ad libitum* access to autoclaved water, standard chow for mice (Nuvilab, Brazil) and paper towels as an environmental enrichment material. The reproduction system used to guarantee heterozygosity was the Poiley method (Lapchik et al., 2009).

### 2.3. Murine model of congenital toxoplasmosis

For mating, one male and two females were cohoused from 5 p.m. to 9 a.m., and day 0.5 of pregnancy (E0.5) was defined as the first observation of a vaginal plug. Females were infected with *T. gondii* tachyzoites by tail vein injections (10^6^ or 10^7^ parasites in 100 μl PBS) at E8.5 or E9.5, which correspond to closure of the neural tube, a critical time in the development of mice (Copp et al., 2003). Control animals received 100 μl PBS by the same route. The mice were euthanized 5 days after infection (E13.5 or E14.5) and had their organs (spleen, liver, brain, and uteri) harvested for further analysis. The mice were frequently monitored after pregnancy detection and until euthanasia, and had their bodies weighed at each of these moments: (1) detection of the vaginal plug, (2) infection, and (3) euthanasia.

### 2.4. Morphometric analysis

Following photodocumentation, the spleens were weighed, and uterine horns were carefully opened to release the fetoplacental units. The resorption rate was calculated for each pregnant dam. The fetoplacental units were dissected, the placental areas were measured, and fetus size was calculated by multiplying the crown-to-rump length (CRL) by the occipital-frontal diameter of the head (OFD) (Cao et al., 2017; Brown et al., 2019). Fragments of each tissue were collected, weighed, and kept at −80°C for further DNA extraction and qPCR analysis.

### 2.5. DNA extraction

At the experimental endpoint, mice were euthanized, and various tissues (spleen, liver, brain, placenta, and fetal head) were harvested and kept at −80°C until further use. Genomic DNA was extracted from 10 to 20 mg of tissue using the High Pure PCR Template Preparation Kit (Roche Diagnostics, Indianapolis, IN, USA). Prior to extraction, tissues were disrupted in 400 μL of tissue lysis buffer using TissueRuptor II homogenizer (QIAGEN, Austin, TX, USA) at full speed for 30 sec. This homogenate was subjected to DNA extraction following the manufacturer’s recommendations. In the last step of the protocol, the DNA was eluted from the silica column in 100 μL of elution buffer, diluted 10 times before evaluation and stored at −20°C for further analysis.

### 2.6. Parasite load quantification by quantitative real-time PCR

Amplification of the 81 bp *T. gondii* fragment of the 529 bp repeat element (RE) DNA was done with SYBR-Green (Life Technologies, Carlsbad, CA, USA) using the specific primers ToxoRE_Fw and ToxoRE_Rv (Table 1; Kasper et al., 2009) both at 300 nM. In addition, specific primers targeting mouse 349 bp β-actin β-actin_Fw and β-actin_Rv (Table 1; Jones et al., 2010), both at 150 nM, were used as an endogenous internal control and for normalization of the parasite load. The standard curve for parasite assessment was made by extracting DNA from 10^5^ parasites/mL of *T. gondii* tachyzoites obtained from mammalian cell cultures spiked into a known weight of different tissues as specified below, making a serial dilution of 1:10 of the eluted DNA in TE buffer, ranging from 10^5^ to 10 parasite equivalents. Standard curves for mouse tissue assessment were made by extracting DNA from 0.2 mg of the placenta, 3.3 mg of the fetal head, 1.1 mg of the spleen, 3.3 mg of the liver, and 2.7 mg of the brain, making a 1:10 serial dilution of the eluted DNA in TE buffer. Real-time PCR reactions were prepared in 20 μL with 2 μL of DNA input and performed in an Applied Biosystems ViiA7 real-time PCR thermocycler (Thermo Fisher Scientific, Waltham, MA, USA) using the following cycling conditions: 50°C for 2 min, 94°C for 10 min followed by 40 cycles at 95°C, and 64°C for 1 min, where fluorescence was collected after each cycle. All samples were run in duplicate, and the threshold was set at 0.02 for both targets. The parasite load was normalized by the amount of tissue assessed by β-actin in each sample.

**TABLE 1:** Primers and standard curve parameters for determination of parasite load in mice infected with *Toxoplasma gondii*.

Target gene	Primer sequences	References	Amplicon length	Tissue	Slope	Intercept	Coefficient of determination (r^2^)	Amplification efficiency (%)
β-Actin	Fw 5′-TGG AAT CCT GTG GCA TCC ATG AAA C-3′ Rv 5′-TAA AAC GCA GCT CAG TAA CAG TCC G-3′	Jones et al., 2010	349 bp	Placenta	−3.45	21.80	0.97	94.58
				Fetal head	−3.54	17.41	0.99	91.40
				Spleen	−3.58	17.01	0.99	90.12
				Liver	−3.96	19.77	0.99	78.69
				Brain	−3.20	35.35	0.99	104.98
ToxoRE	Fw 5′-CAC AGA AGG GAC AGA AGT CGA A-3′ Rv 5′-CAG TCC TGA TAT CTC TCC TCC AAG A-3′	Kasper et al., 2009	81 bp	Placenta	−3.44	36.18	0.98	95.26
				Fetal head	−3.46	35.35	0.99	94.19
				Spleen	−3.46	30.91	0.98	95.07
				Liver	−3.99	33.32	0.99	77.93
				Brain	−3,34	30.89	0.98	99.21

### 2.7. Data analysis

All data were analyzed with GraphPad Prism 7 software (GraphPad, San Diego, CA, USA). At least two independent experiments were performed for each condition of infection. The number of animals or organs analyzed for each condition and experiment is indicated in the figure legends. Each point on the graphs represents data obtained from an individual animal. The results are represented by median ± interquartile range unless otherwise specified. Comparisons between groups were made by one-way or two-way ANOVA with Tukey’s multiple comparisons test, when appropriate. A *p*-value of < 0.05 indicated statistically significant differences.

## 3. Results

### 3.1. Murine model of congenital toxoplasmosis

**FIGURE 1:**
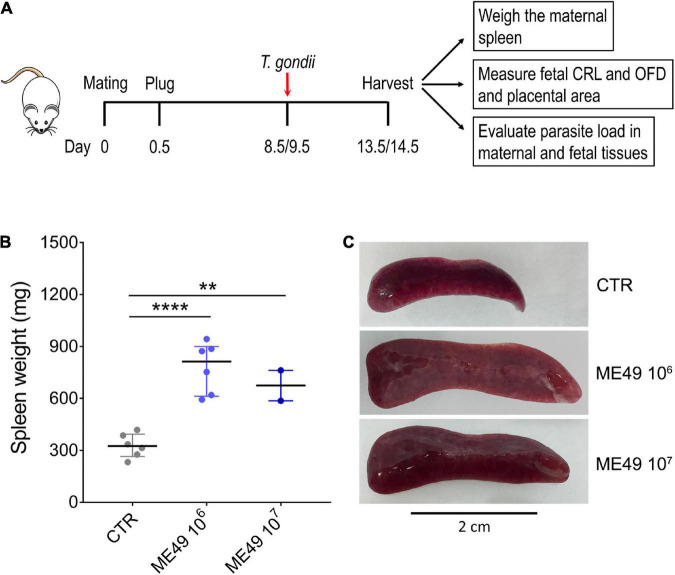
Splenomegaly induced by systemic *T. gondii* infection in outbred Swiss Webster mice. **(A)** Schematic representation of the infection experiment during pregnancy. Pregnant dams were infected intravenously with 10^6^ or 10^7^ tachyzoites (ME49 strain) at E8.5 or E9.5. The mice were euthanized 5 days later, and fetal and maternal tissues were collected for further analysis. CRL, crown-to-rump length; OFD, occipital-frontal diameter. **(B)** The spleen weight of each pregnant dam was evaluated and expressed in mg; *n* = 2–6. CTR, control uninfected group. Each symbol represents data from individual females. Data are presented as median ± interquartile range. One-way ANOVA with Tukey’s multiple comparisons test was employed. **p < 0.01; ****p < 0.0001. **(C)** Representative images of the spleens in each condition.

To address the impact of *Toxoplasma gondii* infection during pregnancy in outbred mice, we established a mouse model of congenital toxoplasmosis by infecting pregnant dams of Swiss Webster (SW) mice with tachyzoites of the *T. gondii* ME49 strain by tail vein injections at E8.5 or E9.5 and harvesting maternal and fetal tissues 5 days later (Figure 1A). Infection induced severe maternal splenomegaly in SW mice, with a 2- to 2.5-fold increase in spleen weight when compared to uninfected controls (Figures 1B, C).

**FIGURE 2:**
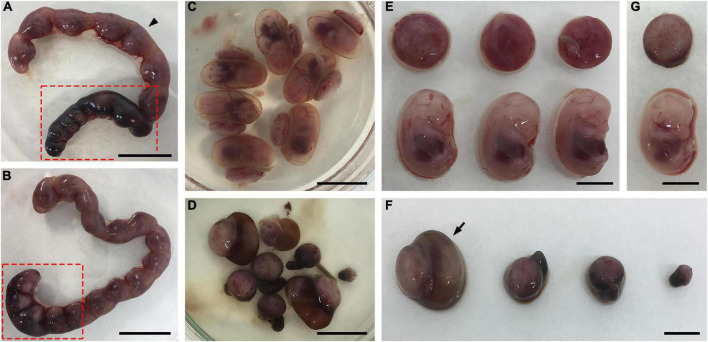
Effects of *T. gondii* infection in outbred Swiss Webster mice during pregnancy. **(A–G)** Pregnant dams were infected with 10^7^ tachyzoites of the ME49 strain at E8.5 or E9.5 and euthanized at E13.5 or E14.5. **(A,B)** Uteri of two different infected mice exhibiting abnormal uterine horns with regions of atrophy and heavy hemorrhage (dark regions highlighted in red box). **(C,D)** Macroscopically normal **(C)** and abnormal **(D)** fetoplacental units were obtained from the superior (arrowhead) and inferior (red box) uterine horns shown in panel **(A)**, respectively. **(E)** Fetuses and placentas from panel **(C)** are apparently healthy. **(F)** The fetoplacental units in panel **(D)** show different degrees of resorptions. **(G)** A fetus taken from the fetoplacental unit indicated with an arrow in panel **(F)** is apparently normal, while its corresponding placenta is pale brown as opposed to the bright red seen in panel **(E)**. The scale bars indicate 2.0 cm **(A,B)**, 1.0 cm **(C,D)**, and 0.5 cm **(E–G)**.

The evaluation of the general aspects of the mothers during the period between the detection of the vaginal plug and euthanasia did not reveal differences in behavior between uninfected and infected dams. Furthermore, body weight gain did not differ between uninfected and infected mothers at three different times: detection of the vaginal plug, infection, and euthanasia (Supplementary Figure 1). However, gross morphological analysis of uteri obtained at E13.5 or E14.5 from *T. gondii*-infected outbred mice revealed abnormal uterine horns with regions of atrophy and extensive hemorrhage (Figures 2A, B), which could indicate the occurrence of a subclinical form of the disease. Interestingly, the fetoplacental units obtained from different regions or different uterine horns from a single infected female had contrasting phenotypes, some of which with apparently normal morphology and others with different degrees of resorptions (Figures 2C–G). It is noteworthy that some placentas had a pale brown coloration as opposed to the normal bright red coloration, although the corresponding fetus was normal sized with an apparently healthy macroscopic appearance (Figure 2G).

**FIGURE 3:**
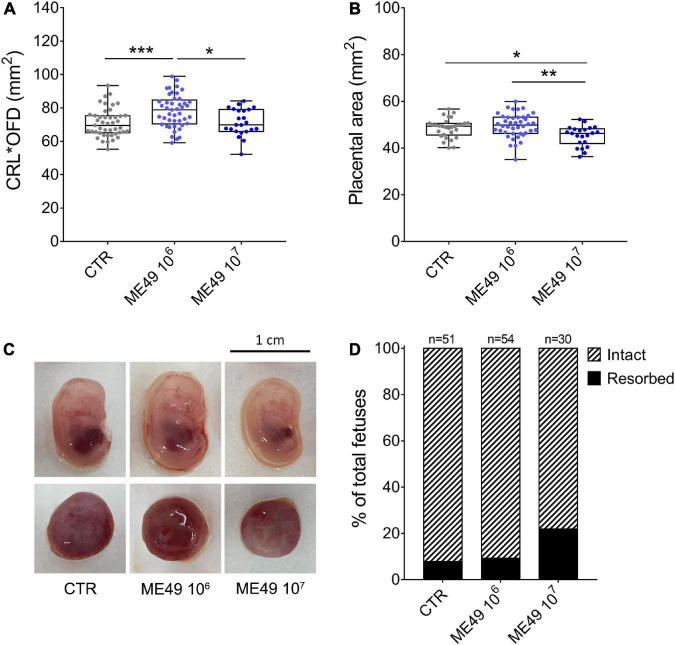
Impact of infection on fetal and placental size and fetal resorption rates during maternal-fetal transmission of *T. gondii* in Swiss Webster mice. Pregnant dams were infected with 10^6^ or 10^7^ tachyzoites (ME49 strain) at E8.5 or E9.5 and euthanized at E13.5 or E14.5. **(A)** Fetal size was calculated by multiplying the crown-to-rump length (CRL) by the occipital-frontal diameter (OFD) and expressed as mm^2^; *n* = 23–45 samples from at least two independent pregnant dams for each condition. **(B)** Placental area was measured and expressed as mm^2^; *n* = 22–42 samples from at least two independent pregnant dams for each condition. Each symbol represents data from individual fetuses **(A)** or placentas **(B)**. Data are presented as median ± interquartile range. One-way ANOVA with Tukey’s multiple comparisons test was employed. *p < 0.05; **p < 0.01; ***p < 0.001. **(C)** Representative images of fetuses and placentas for each condition. **(D)** Resorption rates of uninfected (CTR) or *T. gondii*-infected (ME49 10^6^ and ME49 10^7^) pregnant dams; *n* = 30–54 samples from at least two independent pregnant dams for each condition.

We then investigated the effect of the infection on fetal and placental growth and development by measuring the size of the fetuses (multiplying the crown-to-rump length by the occipital-frontal diameter) and the area of the placentas. Infection of outbred mice with 10^6^ tachyzoites induced a significant increase in fetus size, whereas fetuses obtained from females infected with 10^7^ parasites showed no signs of fetal growth abnormalities compared to fetuses from uninfected dams (Figures 3A, C). Interestingly, placentas obtained from females infected with 10^7^ parasites presented surface areas smaller than those collected from uninfected females or females infected with 10^6^ tachyzoites (Figures 3B, C). Of note, maternal infection did not significantly affect litter size in SW mice (9.4 ± 4.5 in CTR; 9.8 ± 4.9 in ME49 10^6^; 12.5 ± 5.0 in ME49 10^7^, mean ± SD), although the number of resorptions increased from 7.8% in the uninfected group to 21.9% in the group inoculated with 10^7^ tachyzoites (Figure 3D).

**FIGURE 4:**
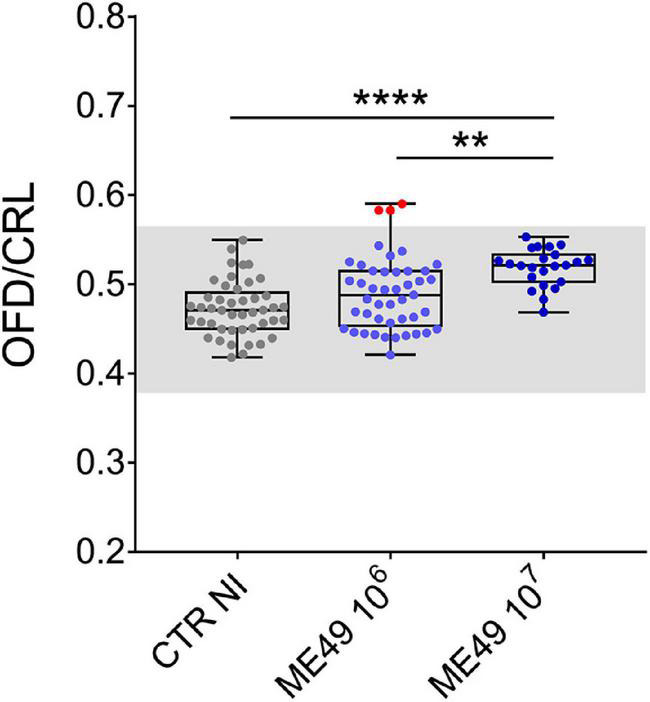
Impact of infection on fetal cephalic proportion relative to body size during maternal-fetal transmission of *T. gondii* in Swiss Webster mice. Data expressed as the ratio between occipital-frontal diameter (OFD) and crown-to-rump length (CRL). The gray region corresponds to the range defined by three SD in relation to the mean values of uninfected controls. Red dots indicate three conceptuses with OFD/CRL values compatible with macrocephaly. *n* = 23–45 samples from at least two independent pregnant dams for each condition. Data are presented as median ± interquartile range. One-way ANOVA with Tukey’s multiple comparisons test was employed. **p < 0.01; ****p < 0.0001.

We also investigated whether the increase in OFD measurements observed in our model could reflect macrocephaly, a sign of congenital cerebral toxoplasmosis (Khan and Khan, 2018). We compared normalized OFD (OFD/CTR) of all fetuses obtained from dams infected or not with *T. gondii* and found that 3 of the 45 fetuses in the group inoculated with 10^6^ tachyzoites presented values above 3 SD in relation to the mean of uninfected controls, values corresponding to clinically relevant macrocephaly (Accogli et al., 2022; Figure 4).

### 3.2. Parasite load by quantitative real time PCR

Assessing the parasite load of pathogens in different tissues is often challenging and requires a well-refined standardization of qPCR to obtain suitable reaction conditions for the best amplification signals. Standardization of qPCR using SYBR Green fluorophore started with primer concentration curves for both β-actin and ToxoRE targets at 64°C annealing/extension temperature in the qPCR cycle, where 150 nM × 150 nM for the β-actin forward and reverse primers and 300 nM × 300 nM for ToxoRE forward and reverse primers showed the best performance, considering Ct values, primer-dimer formation, and reagents cost efficiency (data not shown).

**FIGURE 5:**
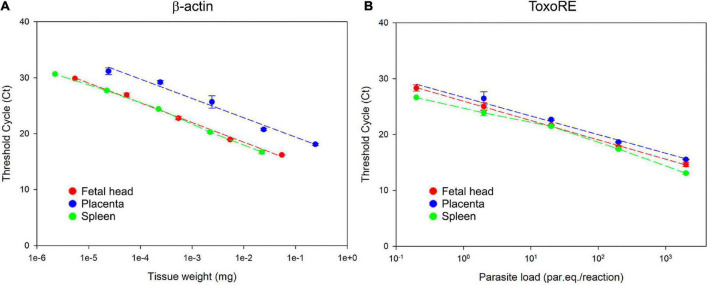
Dynamic range of *T. gondii* detection in fetal (E13.5–14.5) and maternal tissues. **(A)** Standard curve for the β-actin target in fetal head, placenta, and spleen. Ten-fold serial dilutions of different tissues were used to generate the β-actin curves, indicating the linearity of the reaction. **(B)** Standard curve for the ToxoRE target in fetal head, placenta, and spleen. Ten-fold serial dilutions of tachyzoites spiked into tissues were used to generate DNA curves with dynamic ranges of 10^5^ to 10 par.eq./reaction for each tissue.

To achieve and report the experimental conditions and assay characteristics to accurately estimate the parasite load, the MIQE (Minimum Information for Publication of Quantitative Real-Time PCR Experiments) guidelines was followed (Bustin et al., 2009). A robust and accurate qPCR assay generally correlates with high PCR efficiency, and here we evaluated the efficiency for β-actin and ToxoRE targets for each tissue analyzed in this study. The dynamic range over which the reaction was linear (from highest to lowest amount of quantifiable DNA, established by means of a calibration curve) was determined for each target on the standard curve (Figure 5 and Supplementary Figure 2).

It was observed a linearity for the detection of the mice β-actin target from 0.2 mg to 0.2 × 10^–4^mg of placenta, from 3.3 to 3.3 × 10^–4^mg of fetal head and from 1.1 to 1.1 × 10^–4^mg of spleen (Figure 5A). For the ToxoRE target, it was observed a linear detection from 2.0 × 10^3^ to 0.2 parasite par.eq./reaction in placenta, fetal head, and spleen (Figure 5B). For liver and brain, it was observed a linearity from 3.3 to 3.3 × 10^–4^ mg and 2.7 to 2.7 × 10^–4^ mg to the β-actin target, respectively (Supplementary Figure 2A), and for the ToxoRE target, the linearity was the same observed to the other tissues: from 2.0 × 10^3^ to 0.2 parasite par.eq./reaction (Supplementary Figure 2B).

**FIGURE 6:**
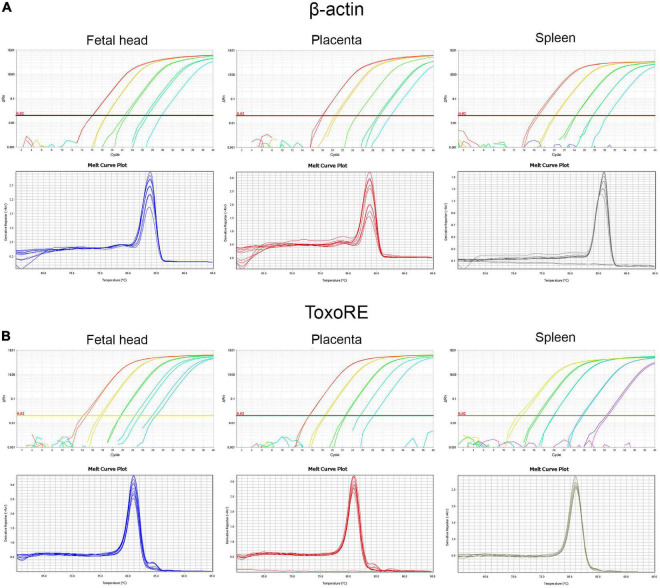
Standardization of qPCR assays to assess the burden of *T. gondii* in fetal (E13.5–14.5) and maternal tissues. Upper panels show representative amplification plot with fluorescent signal magnitude for β-actin **(A)** and ToxoRE **(B)** targets. Lower panels show melting curves indicating the specificity of the reaction, seen through a single peak in each curve for the β-actin **(A)** and ToxoRE **(B)** targets.

Figure 6 shows the amplification plots containing the curves obtained to all dilution points, where the Cts were used to generate the standard curves in fetal head, placenta, and spleen for the β-actin target (Figure 6A–upper panels) and ToxoRE target (Figure 6B–upper panels). Furthermore, melting curves obtained for the same samples are shown for the β-actin target (Figure 6A–lower panels) and ToxoRE target (Figure 6B–lower panels). A single peak was observed in all melting curves, indicating the specificity of the reactions. In contrast, no peak was observed in the Negative Template Control (NTC), indicating the absence of primer-dimers in qPCR. Similarly, Supplementary Figure 2 shows the amplification plots containing the curves obtained for all dilution points, where Cts were used to generate the standard curves in liver and brain for the β-actin target (Supplementary Figure 3A–upper panels) and ToxoRE target (Supplementary Figure 3B–upper panels). In addition, melting curves obtained for the same samples are shown for the β-actin target (Supplementary Figure 3A–lower panels) and ToxoRE target (Supplementary Figure 3B–lower panels). Table 1 shows the qPCR efficiency for β-actin for placenta (94.58%), fetal head (91.40%), spleen (90.12%), liver (78.69%), and brain (104.98%), and for ToxoRE for placenta (95.26%), fetal head (94.19%), spleen (95.07%), liver (77.93%), and brain (99.21%).

**FIGURE 7:**
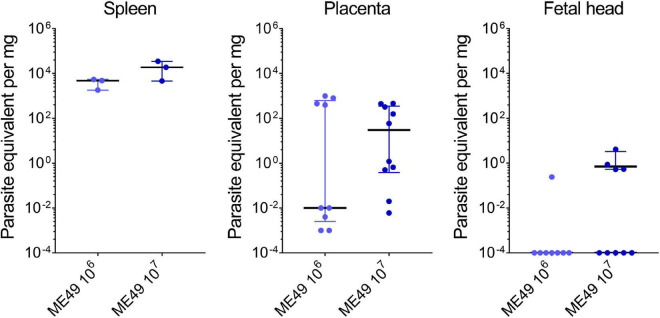
Parasite load in tissues of Swiss Webster mice infected with *T. gondii* during pregnancy. Pregnant dams were infected with 10^6^ or 10^7^ tachyzoites of the *T. gondii* (ME49 strain) at E8.5 or E9.5 and euthanized 5 days later. Maternal and fetal tissues were collected, and the parasite load assessed by qPCR assay. *n* = 3 for each infection condition (for spleens); *n* = 8–10 samples from at least two independent pregnant dams for each infection condition (for placentas and fetal heads). Each symbol represents data from individual mothers or individual placentas and fetuses. Data are presented as median ± interquartile range. Symbols displayed on the x-axis of the last graph represent samples with values below the detection limit of the assay; these samples were not included in the median calculation.

To validate the qPCR assays, we examined the parasite load in maternal and fetal tissues obtained from SW pregnant dams infected with *T. gondii*. The parasite loads in the maternal spleens ranged from 1.80 to 5.47 parasite equivalents (par. eq.) per mg of tissue in the 10^6^ tachyzoite-infected group (4.78 median; 1.80–5.47 interquartile range), and from 4.59 to 34.70 par. eq. per mg of tissue in the group infected with 10^7^ tachyzoites (18.77 median; 4.59–34.70 interquartile range). For placentas, the parasite loads ranged from 0.001 to 982.90 par. eq. per mg of tissue for the ME49 10^6^ group (0.01 median; 0.0025–626.80 interquartile range), and from 0.006 to 460.20 par. eq. per mg of tissue for ME49 10^7^ group (30.04 median; 0.38–352.30 interquartile range). Finally, the parasite load in a single fetal head from the group infected with 10^6^ tachyzoites was 0.24 par. eq. per mg of tissue, while in the group infected with 10^7^ parasites it ranged from 0.52 to 4.11 par. eq. per mg of tissue (0.70 median; 0.53–3.30 interquartile range) (Figure 7). Of note, seven of the eight (87.5%) fetal head samples from the ME49 10^6^ group and five of the nine (55.6%) fetal head samples from the ME49 10^7^ group had undetectable levels of par. eq. in the qPCR assays, indicating a parasite transmission rate for fetuses of 12.5 and 44.4%, respectively.

## 4. Discussion

Here we present a vertical transmission model of *T. gondii* that well reproduces the frequency of parasite transmission and important clinical signs of human congenital toxoplasmosis, in addition to providing a quantitative real-time PCR assay to assess parasite load in maternal and fetal tissues with great sensitivity.

Before reaching the developing fetus, during the acute phase of an orally acquired infection the parasite must spread from the gut enterocytes it encounters first to the placental tissues. For this, they migrate out of the intestinal mucosa, circulating in the lymphatic and blood vessels. However, it is unclear whether *T. gondii* enters the circulation as free tachyzoites or within infected cells, or *via* both pathways (Harker et al., 2015). Several immune cells have been associated with the spread of *T. gondii*, including dendritic cells, lymphocytes, neutrophils, and macrophages. Nevertheless, the relative contribution of each of these leukocytes to parasite dissemination at different stages of infection is unknown (Bhandage and Barragan, 2019). On the other hand, free tachyzoites are found circulating in the peripheral blood of infected humans (Silveira et al., 2011), minipigs (Miranda et al., 2015), and orally infected mice (Unno et al., 2008), supporting the intravenous route as an interesting physiological route for experimental infection.

In our study, inoculation of tachyzoites of the moderately-virulent strain ME49 through the intravenous route with 10^6^ or 10^7^ parasites led to vertical transmissions ratios which largely corresponds to the human transmission rate in the second third of pregnancy (Dunn et al., 1999). The percentage of infected spleens and placentas with the same parasite loads was 100%, confirming that the mothers were successfully infected. The spleen is a key organ in the regulation of the immune response against *T. gondii*, and acute or chronic infection of mice leads to splenomegaly (Chen et al., 2017; Doleski et al., 2017; and present work) and various metabolomic alterations in the organ (including changes in the levels of lipids, hormones, acids, peptides, and other classes of molecules) that can affect the immune response (Chen et al., 2017). In humans, acquired infections with the parasite also lead to splenomegaly (Neves et al., 2009; Abhilash et al., 2013). On the other hand, infiltration of *T. gondii* into the spleen leads to changes in splenic architecture and increased levels of splenocyte apoptosis in mice (Lee et al., 2019). Therefore, detection of parasites in the spleen may help in understanding the outcome of the disease. Our data agree with data obtained by Vargas-Villavicencio et al. (2016b) with BALB/c mice, in which tachyzoite infections at doses of 2.5 × 10^6^ to 10^7^ lead to 100% infection of pregnant mice with the same parasite strain.

The human placenta has two anatomical interfaces that connect maternal and fetal cells: a villous region, where nutrient exchanges between the maternal blood and a large layer of syncytiotrophoblast take place, and the maternal decidua, from where a smaller layer of extravillous trophoblasts bridges the villous region to the uterus (Robbins et al., 2012). Although the mechanism by which *T. gondii* disseminates from mother to fetus is not fully understood, evidence from human placental explant models suggests that the parasite can cross the placental barrier by infecting both the syncytiotrophoblast and the underlying cytotrophoblasts, coming from maternal blood, as well as the extravillous trophoblasts layer, coming from the uterus, from where the parasites reach the fetal circulation (Harker et al., 2015).

Macroscopic examination of the uteri revealed atrophied and hemorrhagic uterine horns with 10^6^ or 10^7^ inoculums, as well as an increased number of fetal resorptions in the larger inoculum. Similar results were found in a congenital transmission model in BALB/c mice inoculated with ME49 parasites intravenously (Vargas-Villavicencio et al., 2016b,^2022^) and in models induced by ME49 cysts administered orally to SW mice (Senegas et al., 2009; Gomes and Barbosa, 2017), where the resorptions were attributed to dilatation of the uterine spiral arteries, apoptosis of placental cells and altered immune responses (Senegas et al., 2009). It is noteworthy that fetuses obtained from different regions or different uterine horns from the same mouse showed distinct phenotypes, some apparently normal and others with variable signs of hemorrhage and/or resorptions. Since each placenta is supplied by two distinct arterial inputs that contribute to the blood flow to fetuses in a fetal positional- and numerically-dependent manner (Raz et al., 2012), and taking into account the crucial role played by the placenta in the control of *T. gondii* infection and vertical transmission (Peyron et al., 2003; Falavigna et al., 2007), the heterogeneity of phenotypes observed here can be explained by a differential distribution of circulating tachyzoites for each fetoplacental unit. Accordingly, in humans, congenital infections of dizygotic but not monozygotic twins lead to clinical manifestations that often differ greatly, and it is not uncommon to find cases of infection in only one twin. This disagreement was attributed, among other factors, to the differential rates of placental vascular supply between the twins, which would lead to different parasite loads (Thapa et al., 2009).

We showed that the placental areas of inoculum 10^7^ were slightly but significantly smaller than those obtained from uninfected SW dams. Accordingly, a slight reduction in placental size area, along with decreased placental weights, was observed in BALB/c mice infected intraperitoneally at day 7 of gestation with 5 × 10^3^ ME49 tachyzoites and analyzed 1 week later (Brito et al., 2020). It is known that the size of the placenta, such as thickness and surface area, reflects its function and efficiency and that a reduction in these measures can lead to abnormal fetal growth (Liu et al., 2021). Therefore, the reduction in placental surface area observed in our model and others can be attributed to vertical *T. gondii* infection, which could explain the fetal growth abnormalities described here and elsewhere (Brito et al., 2020). Of note, we observed that some placentas had a pale brown color instead of the bright red color found in the healthy organ. The brown coloration of organs that receive oxygenated blood, such as the placenta, can be attributed to the presence of oxidized iron atoms in heme molecules usually present in necrotic or hemorrhagic tissues (Piña-Oviedo et al., 2017), which is in line with our observations of hemorrhagic uterine horns after *T. gondii* infection. The presence of regions of necrosis at the maternal-fetal interface has been previously demonstrated in other forms of congenital toxoplasmosis and is associated with the inflammatory immune response to the parasite (Brito et al., 2020).

Among all the symptoms that babies who have acquired congenital toxoplasmosis develop at birth or in the first few months of life are serious neurological problems such as hydrocephalus, microcephaly, and macrocephaly (Khan and Khan, 2018; Del Valle Mojica et al., 2021). We have shown previously that fetuses derived from SW mice inoculated by gavage with tissue cysts of the ME49 strain may show a significant decrease in skull size, characterizing microcephaly (Gomes and Barbosa, 2017). In the present study, fetuses obtained from mothers inoculated with 10^6^ parasites showed an increase in overall size (CRL*OFD), and OFD measurements revealed larger head dimensions in infected fetuses. Therefore, we investigated whether signs of macrocephaly could be present in this model, which could result in the increment of OFD measurements that we observed. In the most rigorous classification of macrocephaly in children, the occipitofrontal circumference must be three standard deviations (SD) above the mean of correctly age- and sex-matched controls to be considered clinically relevant (Accogli et al., 2022). Thus, we compared normalized OFD of all fetuses with attention to the range defined by three SD in relation to the mean values of uninfected controls. It has been well established that children congenitally infected with *T. gondii* present an increased OFD which may reflect ventriculomegaly or other brain malformations (Hutson et al., 2015; Del Valle Mojica et al., 2021; Osman et al., 2021). Interestingly, in our experimental assay the OFD/CRL ratio showed increased values for the infected groups, and 6.7% in the group inoculated with 10^6^ parasites had values above 3 SD, showing evidence of macrocephaly, although none of them had obvious morphological abnormalities. The nature of these changes deserves further investigation.

We also standardized a new cost-effective qPCR method using the SYBR Green system for the quantification of the *T. gondii* parasite in the placenta, fetal head, spleen, brain, and liver of SW mice infected with tachyzoites of the ME49 strain. For this purpose, we used the primers for the repeat element of *T. gondii* genome previously validated using TaqMan system for the assessment of amniotic fluids from patients (Kasper et al., 2009). Although the parasite load in tissue of mice infected with *T. gondii* has been performed previously (Lin et al., 2000; Jauregui et al., 2001; Kasper et al., 2009; Mesquita et al., 2010; Dadimoghaddam et al., 2014; Vargas-Villavicencio et al., 2016b,^2022^; Azadi et al., 2018), this is the first report of extensive validation in various mouse tissues, including placenta and fetal head, as well as maternal spleen, liver and brain. Furthermore, to increase the accuracy of the quantification of the parasite load, we propose the normalization of the parasite equivalents by the amount of tissue, which was also estimated by qPCR targeting the mice β-actin gene. It is noteworthy that the assays reported here reached a sensitivity of 0.2 par.eq./reaction in our linearity assays for fetal head, placenta, and spleen.

In our study, we showed vertical transmission rates of 12.5 and 44.4% for inocula of 10^6^ and 10^7^ tachyzoites, respectively. Accordingly, in a meta-analysis of vertical transmission of *T. gondii* after primary maternal infection, Li et al. (2014) showed a pooled worldwide rate of human congenital transmission of 20%, with incidences increasing progressively from the first to the third trimester of pregnancy. The parasite load we found in the maternal spleen of SW mice was approximately 14 and 130 times higher than in the placenta for the inocula of 10^6^ and 10^7^, respectively. On the other hand, the placenta presented 1,200 and 100 times more parasites than the fetal heads for the inocula of 10^6^ and 10^7^, respectively. Increments of the same order of magnitude were found in BALB/c mice inoculated with 0.25 to 1.0 × 10^7^ ME49 tachyzoites (Vargas-Villavicencio et al., 2016b). In our model, the parasite retention capacity by the placenta was of the order of magnitude of 10^2^ per mg for both parasite loads, indicating that the parasite load is an important factor that can determine whether vertical transmission will occur and to what extent clinical signs will appear. Interestingly, levels of parasite burden in amniotic fluid were associated with clinical outcome in congenital toxoplasmosis in a study conducted in Brazil with women in the first or second trimester of pregnancy (Yamamoto et al., 2017). In that study, 100% of symptomatic cases had parasite loads above the 75th percentile, while all severe cases had parasite loads above the 95th percentile. Therefore, we corroborate previous data, validating our model and reinforce the idea that early prenatal treatment of infected mothers aiming to reduce the parasite load is mandatory for the success of the prevention of vertical transmission, impacting on clinical signs and long-term sequelae.

In conclusion, we present here a model of congenital toxoplasmosis that recapitulates important aspects of the human disease, such as macrocephaly. This model could be further used for therapeutic purposes with approaches aimed at preventing mother-to-fetus transmission. We also standardized and validated a qPCR method using SYBR Green to detect parasite in various maternal and fetal tissues, paving the way to investigate with high precision and sensitivity the potential for vertical transmission in mice. Considering that the qPCR protocol described here can be applied regardless of the route chosen to inoculate the parasite, this tool can be used to compare different experimental models of congenital toxoplasmosis (e.g., intravenous inoculation of tachyzoites versus oral administration of tissue cysts). In future work, it will be interesting to correlate the inoculum given by different routes both with the parasite load in maternal tissues and with the mother-to-fetus transmission rate of *T. gondii*.

## Data availability statement

The raw data supporting the conclusions of this article will be made available by the authors, without undue reservation.

## Ethics statement

This study was carried out in strict compliance with the recommendations of the Brazilian National Council for the Control of Animal Experimentation (CONCEA). All procedures were approved by the Institution’s Ethics Committee for the Use of Animals (CEUA/IOC, license numbers: L-040/2018-A1 and L-042/2018-A1).

## Author contributions

RM conceived the work and drafted the manuscript. RM, JS, and OM worked on the study design. PF and OM wrote the quantitative real-time PCR part of the manuscript. JS, PF, BF, and RM performed the bench work. JS, PF, OM, and RM analyzed the data. HB and RM-B critically reviewed the work and made many contributions to the manuscript discussion. All authors contributed to manuscript revision, read, and approved the submitted version.
