# Supplementary material for: Establishment of a murine model of congenital toxoplasmosis and validation of a qPCR assay to assess the parasite load in maternal and fetal tissues
Source: Front Microbiol. 2023 Feb 27;14:1124378. doi: 10.3389/fmicb.2023.1124378 (PMC10009190; doi:10.3389/fmicb.2023.1124378)
Supplement: Supplementary file 1 [file Data_Sheet_1.PDF]

frontiers

Supplementary Material

Establishment of a murine model of congenital toxoplasmosis and validation of a qPCR assay to assess the parasite load in maternal and fetal tissues

Jéssica S. Souza, Priscila S. G. Farani, Beatriz I. S. Ferreira, Helene S. Barbosa, Rubem F. S. Menna-Barreto, Otacilio C. Moreira*, Rafael M. Mariante*

*** Correspondence:**
Rafael M. Mariante
rafael.mariante@ioc.fiocruz.br; rafaelmariante@gmail.com

Otacilio C. Moreira
otacilio@ioc.fiocruz.br

Supplementary Figures

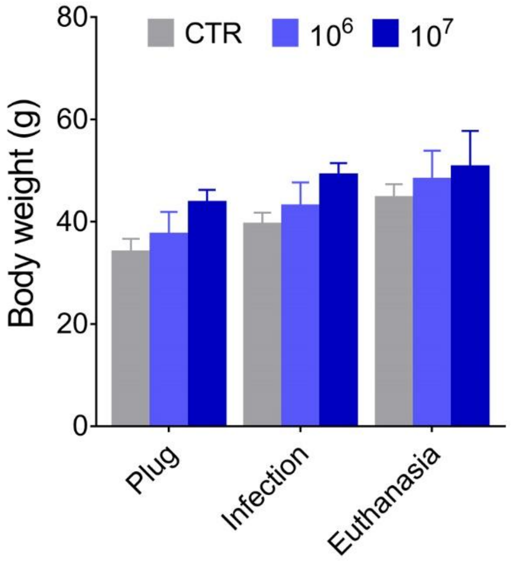

SUPPLEMENTARY FIGURE 1 | Body weight of pregnant mice at the time of vaginal plug detection, infection with 10^6 or 10^7 tachyzoites of *T. gondii* (E8.5 or E9.5), and euthanasia (E13.5 or E14.5). Two-way ANOVA with Tukey's multiple comparisons test was employed, but no statistical differences were observed between uninfected or infected dams at each moment analyzed; n = 2–6. CTR = control uninfected group. Data are presented as mean ± standard deviation.

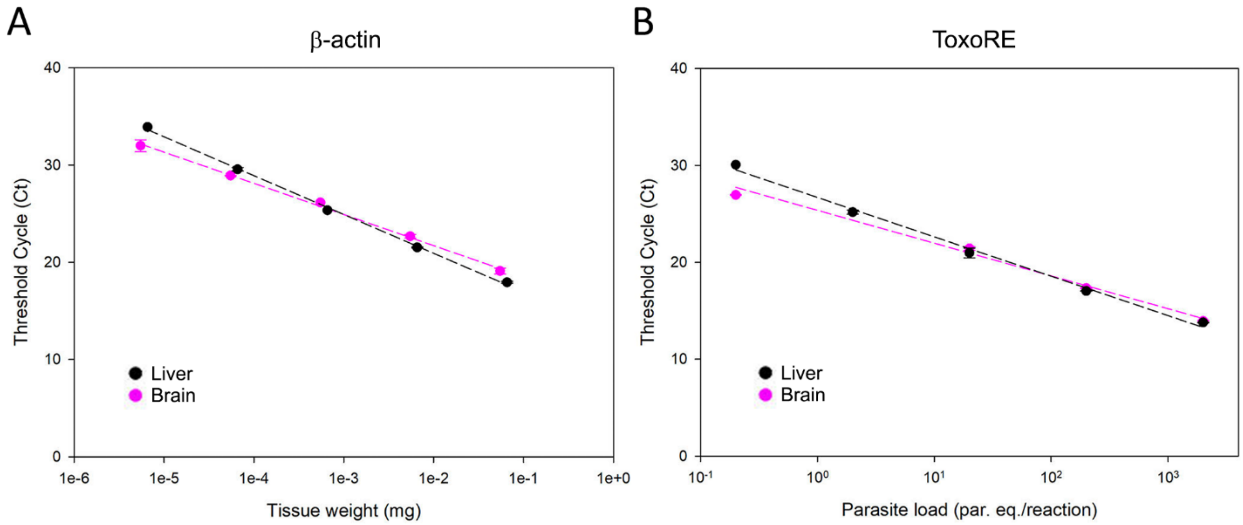

SUPPLEMENTARY FIGURE 2 | Dynamic range of *T. gondii* detection in maternal tissues. **(A)** Standard curve for the β-actin target in liver and brain. Ten-fold serial dilutions of each tissue were used to generate the β-actin curves, indicating the linearity of the reaction. **(B)** Standard curve for the target ToxoRE in liver and brain. Ten-fold serial dilutions of tachyzoites spiked into tissues were used to generate DNA curves with dynamic ranges of 10^5 to 10 parasite par.eq./reaction for each tissue.

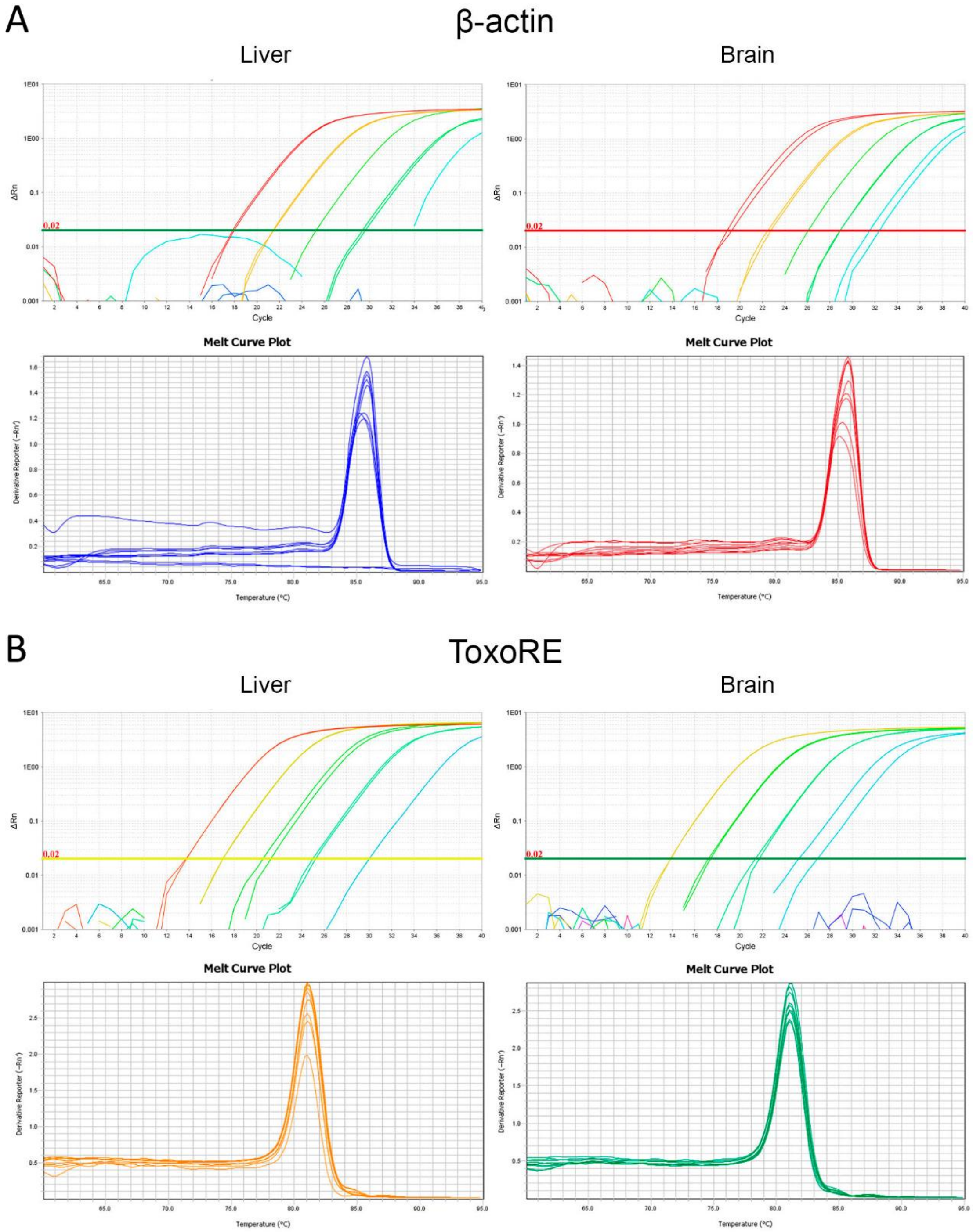

SUPPLEMENTARY FIGURE 3 | Standardization of qPCR assays to assess the burden of the *T. gondii* in liver and brain. **Upper panels** show representative amplification plot with fluorescent signal magnitude for β-actin **(A)** and ToxoRE **(B)** targets. **Lower panels** show melting curves indicating the specificity of the reaction, seen through a single peak in each curve for the β-actin **(A)** and ToxoRE **(B)** targets.
